# Machine learning assists in increasing the time resolution of X-ray computed tomography applied to mineral precipitation in porous media

**DOI:** 10.1038/s41598-023-37523-0

**Published:** 2023-06-29

**Authors:** Dongwon Lee, Felix Weinhardt, Johannes Hommel, Joseph Piotrowski, Holger Class, Holger Steeb

**Affiliations:** 1 Institute of Applied Mechanics (CE), University of Stuttgart, Pfaffenwaldring 7, 70569 Stuttgart, Germany; 2 Institute for Modelling Hydraulic and Environmental Systems, University of Stuttgart, Pfaffenwaldring 61, 70569 Stuttgart, Germany; 3 Agrosphere (IBG-3), Institute of Bio- and Geosciences, Forschungszentrum Jülich, 52425 Jülich, Germany; 4 SC SimTech, University of Stuttgart, Pfaffenwaldring 5, 70569 Stuttgart, Germany

**Keywords:** Civil engineering, Environmental sciences, Solid Earth sciences

## Abstract

Many subsurface engineering technologies or natural processes cause porous medium properties, such as porosity or permeability, to evolve in time. Studying and understanding such processes on the pore scale is strongly aided by visualizing the details of geometric and morphological changes in the pores. For realistic 3D porous media, X-Ray Computed Tomography (XRCT) is the method of choice for visualization. However, the necessary high spatial resolution requires either access to limited high-energy synchrotron facilities or data acquisition times which are considerably longer (e.g. hours) than the time scales of the processes causing the pore geometry change (e.g. minutes). Thus, so far, conventional benchtop XRCT technologies are often too slow to allow for studying dynamic processes. Interrupting experiments for performing XRCT scans is also in many instances no viable approach. We propose a novel workflow for investigating dynamic precipitation processes in porous media systems in 3D using a conventional XRCT technology. Our workflow is based on limiting the data acquisition time by reducing the number of projections and enhancing the lower-quality reconstructed images using machine-learning algorithms trained on images reconstructed from high-quality initial- and final-stage scans. We apply the proposed workflow to induced carbonate precipitation within a porous-media sample of sintered glass-beads. So we were able to increase the temporal resolution sufficiently to study the temporal evolution of the precipitate accumulation using an available benchtop XRCT device.

## Introduction

Subsurface reservoirs are increasingly used for fluid storage, and many of the applied technologies are linked to the production or storage of energy, often controversially discussed in the society^1^. Many recent subsurface activities aim at storing energy in the form of compressed air, CH$_4$, or H$_2$ to cope with unstable production of renewable sources like wind and solar^2^, or directly combating climate change by the sequestration of CO$_2$^3^.

Fluids stored in the subsurface may in some cases leak from the target reservoir. This reduces storage efficiency and can potentially pose a threat to the environment or other subsurface utilization^1^. Induced carbonate precipitation (ICP), induced, for example, enzymatically or microbially (E/MICP), is an emerging technology to mitigate such leakages that has been shown to be effective also in field experiments^4–8^.

ICP has many additional applications such as ground reinforcement, remediation, erosion control, and more^9–12^. Field- or large-scale applications of ICP with the aim of soil property modification have also been increasingly conducted in the past years^13–18^. Although the changes in permeability are of minor interest in applications for ground reinforcement, there may be sufficient precipitation to affect flow paths and thereby the transport of reactants at a larger scale, making accurate porosity-permeability relations important even for non-leakage-mitigation applications of ICP. Enzymatically induced carbonate precipitation (EICP) is one of the possible methods of achieving ICP, but many other methods of inducing carbonate precipitation exist^19^. During EICP, the enzyme urease catalyzes the hydrolysis reaction of urea ($\mathrm{(NH_2)_2CO}$) into ammonia ($\mathrm{NH_{3}}$) and carbon dioxide ($\mathrm{CO_2}$). This reaction increases pH as aqueous solutions of ammonia become alkaline. A more and more alkaline solution results in increased concentrations of carbonate ($\mathrm{CO_{3}^{2-}}$), as it is the dominant species of inorganic carbon at high pH conditions. In the presence of calcium ($\mathrm{Ca^{2+}}$), high carbonate concentrations result in the precipitation of calcium carbonate ($\mathrm{CaCO_{3}}$). The overall EICP reaction is:

1 $$\begin{aligned} \mathrm{(NH_2)_2CO} + 2\mathrm{H_2O} + \mathrm{Ca^{2+}} \longrightarrow 2\mathrm{NH_{4}^{+}} + \mathrm{CaCO_{3}}\downarrow \end{aligned}$$

More details and background to our EICP studies and the experimental workflow can be found in e.g. Refs.^20–22^.

To design and evaluate ICP applications or to investigate the applicability of ICP more generally, numerical models have been developed and applied to real and general leakage mitigation scenarios^23–25^. For numerical models concerned with leakage mitigation on a field, i.e. Darcy scale, the prediction of effective porous medium properties, such as porosity and, in particular, permeability is crucial, since successful leakage mitigation equates to sufficiently reduced permeability so that leakage is prevented. However, many numerical models for ICP only consider simplistic porosity-permeability relations^8,26–29^. A widely used approach in experimental and numerical investigations of reactive transport is the simple power-law relation^30^:

2 $$\begin{aligned} k/k_0 = (\phi /\phi _0)^\eta , \end{aligned}$$

with the intrinsic permeability, *k*, and the porosity, $\phi$, linked by the exponent $\eta$. The subscript, “0”, indicates the initial values respectively. According to Hommel et al.^30^, a power law can be considered as a default first choice when modeling transport through an evolving porous medium, since it only has one parameter, $\eta$, that can be fitted to observations and, thus, makes resulting porosity-permeability relations easily comparable to other studies by comparing the determined exponents. The Kozeny-Carman type approach was not considered in this work due to the potential increase in complexity (interested readers can refer to Supplementary Information).

An important step to improve the predictive capacity and reliability of numerical models on the field scale is the development of process- and porous-medium specific porosity-permeability relations. To investigate such relations in laboratory experiments, a major challenge is to measure both pressure and pore morphology change at a high temporal resolution while ICP occurs in a sample. (The estimation of characteristic time of ICP at our conditions is approximately 5000 s, interested readers can refer to Supplementary Information).

In optical transparent 2D systems, tracking the geometry change with high temporal resolution is fairly straightforward with conventional light microscopy as described in previous (microfluidic) studies^31–34^. Moreover, there are many other possibilities to resolve changes in geometry over time and to study fluid dynamics. By utilizing transparent 2D systems, one can obtain the velocity profile of a fluid during dynamics via particle image velocimetry (PIV). Modern confocal microscope devices provide limited 3D information during fluid flow. Both confocal microscopy and particle image velocimetry (PIV) support understanding of transport in porous media and adopted to investigate dynamics in 2D system^35,36^.

While 2D studies have significant value in improving process understanding, the neglected third dimension still leaves uncertainties with respect to the results from such 2D studies, whether or not they can be applied to realistic, 3D subsurface settings. One of pronounced methods to visualize 3D structures is magnetic resonance imaging (MRI). MRI is widely used in the medical field due to its non-invasive nature, and although it is conventionally known for its low spatial resolution, modern devices can resolve structures as small as 10-20 $\mu$[m]^37^. However, achieving accurate imaging requires a trade-off between spatial and temporal resolutions, and more data acquisition is necessary. In specific occasions, a reported temporal resolution can be up to approximately 100 [ms] with a spatial resolution of 1 [mm] (interested readers can refer to the work of Nayak et al.^38^). Such systems have been adopted to visualize dynamics in 3D porous media^39–41^, but they have certain limitations due to their trade-off nature.

For reducing uncertainties in determining porosity-permeability relations with respect to dimensionality, realistic pore geometries, and solid mineral composition as well as solid surfaces of intransparent 3D samples, like rock cores, need to be investigated and, thus, imaging needs to be conducted by X-Ray Computed Tomography (XRCT). The scanning time of conventional XRCT devices is characterized by long acquisition time (several minutes to hours). Some modern lab-based XRCT devices allow for fast imaging (several seconds to minutes) due to the rapid development of hardware components^42,43^. While those components are a limiting factor for fast scanning, we focused on a cost-effective workflow that significantly shortens conventional XRCT acquisition time to enable a higher temporal resolution of the geometry data during precipitation.

Recently, XRCT has been robustly adopted as a non-invasive method to explore physical phenomena in various fields^44–48^. The method provides a full 3D geometry information of the target which allows an intuitive overview. A typical lab-based XRCT is the standard cone-beam setup where it has a trade-off between spatial resolution and data acquisition time^49^. This trade-off is mainly triggered by the relation between source energy capacity and focal size. For example, reducing a focal spot size is required in order to achieve a higher spatial resolution. However, using reduced focal spot size leads to limiting the amount of energy that can be emitted from X-ray source, which in turn increases the scanning time^50^. As a result, it requires longer exposure time, meaning longer data acquisition time, to dose sufficient X-ray in order to achieve accurate images^51^. In addition, acquiring many numbers of projections from different angles of the target is essential in order to reconstruct precisely the 3D geometry of the target with XRCT^52^. Due to the combination of exposure time and the required number of projections, XRCT often suffers from long data acquisition time, which limits accordingly the temporal resolution to explore full 3D dynamic phenomena.

Observing relatively fast process behaviors, below the time resolution of conventional lab-based XRCT, such as solute transport, dissolution of minerals, gas bubble dynamics or flow processes of immiscible fluids, is so far available at advanced $\mu$XRCT setups^53–55^ and synchrotron facilities^56–61^.

In an advanced $\mu$XRCT setup such as in the work of Bultreys et al.^53^ and Offenwert et al.^54^, the authors showed solute transport in porous media with very high temporal resolution. The authors were able to achieve scanning time down to 12 [s] with the help of limited number of projections and binning which involves combining adjacent pixels. Binning is beneficial to increase signal-to-noise ratio and therefore shorten the exposure time. Additionally, the authors adopted “smooth shooting” strategy, where they obtained projections from different angles while continuously rotating, which helped to further boost the speed of the entire scanning process. In the work of Dewanckele et al.^55^, the authors were even able to achieve faster scanning time (9.6 [s]) by using an improved setup.

With several orders of magnitude higher flux of X-ray, it is possible to acquire 3D data sets in sub-seconds with a very high spatial resolution (below micrometer resolution) in a synchrotron facility^62,63^. In addition, due to the synchroton beam’s monochromatic nature, a further evaluation of the resulting image is simpler compared to conventional lab-based XRCT imaging, which utilizes a polychromatic beam. This causes the artefact of beam hardening^64^. With the help of such an advanced means of observation, aforementioned studies have brought a huge benefit to understand physics during fast processes, resulting in an intense scientific interest. However, despite of such advantages, the opportunity to utilize synchrotron facilities is very rare and proposal-based. The main reason is the low number of synchroton facilities compared to scientific demands. In addition, the associated cost of conducting experiments in such facilities is very large^56^.

Instead of relying on hardware improvements or advanced synchroton facilities, there have been also active studies on software-based imaging techniques in order to improve the speed of data acquisition^65–68^. Commonly, those studies rely on a low-dose strategy, which is beneficial to optimize data acquisition time by avoiding a full-/ideal data acquisition scenario. Also, the aim of these studies is to enhance the image quality degraded as a trade-off by adopting a low-dose strategy.

The low-dose strategy could be classified in two categories: ($1^{\text {st}}$) reducing the X-ray dosage^67,69^ and ($2^{\text {nd}}$) limiting the number of projections (sparse-view)^70–72^. In the first approach, the amount of dosage can be reduced by decreasing the voltage or the flux of the beam source as well as restricting the exposure time. This plays a very crucial role, especially in the field of medical imaging^73^, in order to minimize a radiation exposure to patients. Besides, in the study of biological cells^69^ or polymers^74^, this approach has a huge impact since the high energy beam is ought to be avoided for not damaging the target sample. In the second approach, one can utilize only a limited number of projections (sparse-views) to reconstruct an adequately good 3D image stack. By taking only a few snapshots from several different angles, optimizing the scanning time and reducing the total amount of X-ray dosage during scanning is possible. This approach aims at achieving qualitatively equivalent 3D imaging results compared to fully-sampled data, but from under-sampled data. This sparse-view approach is very promising not only in the medical imaging field^70^ but also in industrial applications^75^. This is because the access angles of X-ray observation can be limited in industry applications such as the investigation of aircraft wings due to the size of the target^76^. In addition, even in other imaging techniques such as photoacoustic imaging^77^, where access from many different angles on patient’s skin is restricted, this approach could be advantageous. In spite of the desirable benefits in the aforementioned aspects and especially in the reduced scanning time, the major problem of such low-dose approaches is that the resulting images often suffer from severely decreased quality. This is because of the innate trait of XRCT which needs to collect X-rays passing through the target. Generally, imaging with a long exposure time in combination with a sufficient amount of energy is likely to provide a better and stable “noise-clean” result since more X-rays are collected at the detector statistically^78^. In a sparse-view approach, the lack of information due to sparse angular sampling is therefore the challenging problem for the reconstruction of good quality 3D data sets.

### Conventional reconstruction methods

In order to tackle such issues, there have been many studies to improve reconstruction methods which are capable of dealing with low-dosed tomographic data^67,71,72,79^. The authors suggested iterative methods to reconstruct images instead of using the FBP (Filtered Back Projection) method which is a conventionally used analytic reconstruction approach^72^. In the FBP approach, the reconstruction of images is dealt with as an inversion problem, meaning the method demands sufficient number of full-span projections to reconstruct “good image stacks”^80^. Due to this reason, for a sparse-view problem, reconstruction with FBP often results in serious streaking artefacts which makes further image evaluation difficult^67^. Unlike to FBP, in iterative approaches, the methods seek a proper solution, meaning “good image stacks” for a sparse-view problem by adopting multiple iteration steps^71^. It repeatedly updates the value within each voxel while minimizing the difference between a weighted projection, which is computed based on Siddon’s algorithm^81^, and a measured one^72^. The Siddon’s algorithm quickly calculates how an X-ray beam passes through a target sample by dividing the beam into line segments and estimating the attenuation of the beam by those lengths as it intersects with voxels. In the work of Beister et al.^71^, they authors demonstrated that this approach was able to suppress noise and streaking artefacts effectively in sparse-view reconstruction comparing to the conventional FBP method. However, due to the high computational cost caused by iteration steps which should be performed until it reaches a good quality agreement, the time and computational efficiency of such methods are often challenging^72,79^.

### Machine learning based methods

In recent years, machine-learning (ML) based schemes have emerged as a very powerful tool to compensate the quality drop issue of low-dosed XRCT images, as a different approach^70,76,82,83^.

With the support of the well-known flexibility of ML schemes where the ML model can be trained in a data-driven fashion, the quality degradation issue of low-dose XRCT has been challenged in mainly two aspects: advanced reconstruction methods^82,84^ and enhancing the quality of images after temporary reconstruction^70,77,83,85^.

#### Machine learning based methods: reconstruction

In an advanced reconstruction method approach, Pelt et al.^82^ proposed their reconstruction ML model combined with FBP implicitly. By applying FBP to given projections with different weights, the model tries to optimize those weights which could provide an ideal output (reconstructed image). In their work, they demonstrated that their model outperformed conventional FBP and iterative approaches. In addition, they claimed that the model was computationally efficient compared to conventional iterative approaches due to its FBP based trait. Instead of relying on an innate conventional algorithm, Zhu et al.^84^ suggested the AUTOMAP model fully driven by data which automated the reconstruction process. By adopting fully connected layers in their model architecture, which gives a strong link between inputs and outputs while minimizing data loss, they designed their model to be capable of mapping the reconstruction image from the acquired projections. In their work, they showed that the model was able to produce a noise-degraded output compared to conventional methods in sparse-view problems. In spite of fascinating results, which showed the outstanding performances against sparse-view data, this type of advanced reconstruction methods tends to require large computational memory for such a complex machine learning architecture. Especially, the fully connected layers which are essential to lead the reconstruction procedure in a data-driven fashion, can potentially result in a huge number of trainable parameters demanding a large amount of memory^86^. In addition, the required computational memory increases exponentially corresponding to the number and size of the input projections.

#### Machine learning based methods: refining reconstructed image

In the studies of enhancing reconstructed image quality, as another approach, Wolterink et al.^83^ proposed the GAN (Generative Adversarial Networks) model targeting low-dose XRCT data. The authors suggested a combination model of discriminator CNN (Convolutional Neural Network) and generator CNN. The generator CNN, which mimics high-dose XRCT images, creates noise-degraded images from a given low-dose image by employing regression. Sequentially, either an output of generator CNN or actual high-dose image is given to the discriminator model, which determines whether the input is an actual high-dose image or an artificial one. Further, the result of the discriminator model was employed as an adversarial feedback to the generator model. By training their model in this fashion, the authors showed that their generator was able to provide a noise-suppressed and realistic image, comparable to the reference routine-dose XRCT data. However, the training instability of such a model and the inherent mode collapsing problem, which over-simplifies varieties of data, often limits its usability and requires a careful study^87^.

As convolutional neural networks (CNN) showed impressive performance in various image processing applications^88–90^, this type of architecture also has been studied widely in order to tackle noise and artefact issues of low-dose XRCT data^70,76,85^. In the work of Jin et al.^70^, the authors proposed FBPConvNet where its architecture is based on the famous U-net approach^91^, which is of sequential and multiple de-/convolutional layers. By training their model with a combination of full-view and sparse-view FBP reconstructed images, the model was able to produce a qualitatively full-view like image with a given under-sampled reconstruction result. In the work of Wang et al.^76^, with the same type of U-net model called SARTConvNet, the authors trained their model with reconstructed images from the SART (simultaneous algebraic reconstruction technique) algorithm^92^, which is an iterative reconstruction method, instead of using high-dose FBP reconstructed images. In this way, they were able to produce a slightly better signal-to-noise ratio of the reconstruction result than FBPConvNet from sparse-view data. Furthermore, inspired by such promising results, the approach of enhancing reconstructed images with CNN type of model was broadly used also in other applications such as OCT (Optical Coherence Tomography)^93^ and photoacoustic imaging^77^, in order to resolve the sparse-view problem.

Fast scanning techniques that use binning to enhance signal-to-noise ratio with shorter exposure time often lead to degraded image resolution^43^. To address this issue, studies have explored the use of different machine learning models, including ResNet. Wang et al.^94^ employed a ResNet-based model to enhance resolution by training it on a combination of high and low-resolution images synthetically downsampled from high-resolution images. ResNet is a popular model architecture in computer vision known for its effective use of residual connections to enable direct flow of information through the network.

In a different study, Tang et al.^95^ used CycleGAN to address the noise issue caused by synchrotron radiation’s low-intensity signal and short exposure time. They trained their model with a combination of common $\mu$XRCT and synchrotron data, allowing CycleGAN to transfer the noise from synchrotron radiation into the common $\mu$XRCT data. This technique enabled them to segment synchrotron data while suppressing the noise, utilizing CycleGAN’s ability to learn the mapping between different image domains^95^.

### Our method

Considering the aforementioned pros and cons of previous works, we propose our time-resolved XRCT workflow which allows us to observe the EICP process with time resolution in 3D, only with the help of a conventional lab-based XRCT device. We were able to reduce the data acquisition time from three hours to approximately six minutes during the EICP process by avoiding a full data acquisition scenario (high-dose). We considered this reduced scanning time of approximately 6 min to be fast enough to assume steady-state conditions during scanning. The corresponding noise and artefacts, caused by the low-dose strategy, were successfully suppressed by adopting the 3D U-net architecture using the full 3D spatial information. The adopted model was trained with reconstructed low-dose images and their corresponding reconstructed high-dose images, which were acquired after the EICP process. The FBP method, which is simple and, thus, computationally effective was used to reconstruct the images. Based on the refined data, we were able to observe the decreasing tendency of porosity triggered by the calcium carbonate precipitation. In addition, the pressure drop between the in- and outlet, which was measured in parallel during EICP, showed an increasing tendency accordingly. The estimated porosity from the refined scanning data was well-bounded within the porosity range of the high fidelity data (full acquired data), which was obtained before (initial) and after (final) the EICP process. In addition, the estimated porosities result in power-law exponents fitted for the porosity-permeability relation in the range of previously reported values for mineral precipitation in porous media^30^.

## Material and methods

### Sample and reactive solution preparation

#### Preparation of the columns

**Figure 1:**
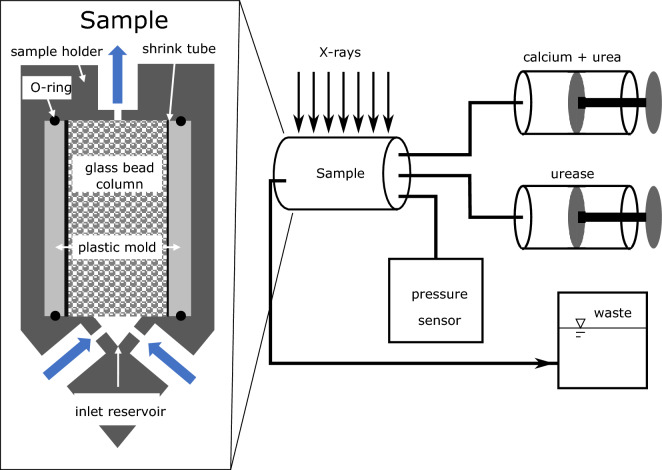
Sketch of the EICP setup as used in this study (right) and a detailed sketch of the sample holder (left).

The sample columns with a diameter *d* = 5 mm and the length $L = 10$ mm were sintered from borosilicate glass beads of $180 \mu \textrm{m}$ mean diameter. After sintering, the columns were wrapped in a shrink tube and fixed into a cylindrical, 3D-printed plastic mold by epoxy resin. This was done to ensure consistent outer dimensions of each sample and to provide a flat surface for sealing potential bypassing flow around the sample using o-ring seals in the sample holder (see Fig. 1). In this investigation, glass bead column samples were used that had been mineralized in previous experiments, after dissolving the previous precipitation by submersion of the columns in hydrochloric acid over night. Additionally, the samples were put in hydrochloric acid under vacuum over night to remove the gas created during the dissolution of the carbonates allowing the acid to contact the carbonates and a more efficient dissolution. After dissolution, the samples were flushed with deionised water and dried over night in an oven at $68^\circ$C.With the aforementioned procedure, two borosilicate glass beads columns were prepared and named BGC1 and BGC2.

#### Preparation of reactive solutions

Two reactive solutions were prepared following the workflow described in^21^: For preparing Solution 1, urea (MERCK^©^) and calcium chloride were dissolved in deionized water at equimolar concentrations of 1/3 $\frac{mol}{L}$. For Solution 2, the enzyme urease was extracted from jack-bean meal (Sigma Aldrich^©^): A suspension of jack-bean meal at a concentration of 5 $\frac{g}{L}$ was stirred at 8 $^\circ$C for 17 h and subsequently filtered twice through a cellulose membrane with a filter size of 0.45$\mathrm {\mu m}$ before use in experiments.

### Experimental setup and procedure

The setup for EICP is sketched in Fig. 1. The glass-bead column was placed in a sample holder with three inlets as in the work of Hommel et al.^96^. Two inlets were connected to the two syringe pumps respectively (mid pressure pumps, type neMESYS 100N and 25ml glass syringes from CETONI GmbH, Korbussen, Germany). One syringe was filled with Solution 1, containing calcium chloride and urea, and the other one with Solution 2, containing urease. The third inlet was connected to a pressure sensor with a maximum pressure of 1 bar (type MPS2 from Elveflow, Paris, France). The outlet was connected to a waste container with a controlled constant head (back-pressure); the diameter of the outlet tube was large enough (1.6 mm) to neglect the pressure drop along its length. Therefore, the pressure measured at the inlet subtracted by the constant head pressure of the outlet can be assumed to be the pressure drop along the column at a given flow rate.

Similar to the experimental procedure presented in^21^, the experiment can be subdivided into three stages: (a) initial permeability measurement, (b) continuous injection of reactive solution, and (c) final permeability measurement, with the ambient temperature being 27 $^\circ$C.

At first, the permeability was determined by applying different flow rates up to 0.6 $\frac{mL}{s}$ with deionized water only (Stage a). The measured pressure at the inlet subtracted by the constant head pressure of the outlet corresponds to the pressure drop of the cell caused by the flow through the column. Based on these measurements, the intrinsic permeability, *k*, of the porous domain can then be determined by rearranging Darcy’s Law^21^:

3 $$\begin{aligned} k = - \frac{\mu L_{column} Q}{A \Delta p}, \end{aligned}$$

with *A* being the cross-sectional area ($A = \pi \frac{d_{column}^2}{4}$) of the column, *Q* the applied flow rate and $\mu$ the dynamic viscosity of the fluid, which for stage (a) and (c) is deionized water at 27 $^\circ C$ ($\mu = 0.85 \textrm{mPas}$). The mineralization of the glass-bead column (Stage b) was promoted by co-injecting both solutions, urease as well as urea and calcium chloride, into the glass-bead columns at a constant flow rate of $0.5~\frac{\mu l}{s}$ each. Note, that there is a designed reservoir in the inlet of the sample holder which helps to mix the solutions during the injection. A total of 24  ml of each solution was injected, resulting in a continuous injection during 13.33 h. The flow rate was chosen to ensure creeping flow conditions with Reynolds Number (Re) $<1$. During the entire mineralization process, the inlet pressure was monitored. Using the reasonable assumption of constant viscosity during the co-injection of the reaction solutions, the permeability, normalized by the initial permeability $k_0$, ($k/k_0$) can be calculated as the reciprocal of the normalized pressure drop ($k/k_0 = \Delta p_0 / \Delta p$)^21^.

After mineralization, the system was flushed with deionized water, in order to replace the reactive solutions inside the column and to avoid further precipitation. Finally, another permeability estimation was conducted by applying flow rates from 0.05 to 0.2 $\frac{mL}{s}$ with deionized water, while measuring the corresponding inlet pressure (stage c).

### $\mu$XRCT and image acquisition

In this study, the XRCT data is acquired with a modular and open micro X-ray Computed Tomography ($\mu$XRCT) system^97^ at the Porous Media Lab (PML) conducted by the Institute of Applied Mechanics (CE) of the University of Stuttgart. The X-ray source was operated with a power of $14.3\textrm{W}$, with an acceleration voltage of $130\textrm{kV}$ and an acceleration current of $110\mu \textrm{A}$. The sample was placed on rotational stage and imaged with the CMOS flat panel detector Dexela 1512NDT (PerkinElmer, Inc., Waltham, MA, USA). Detailed specifications of the detector and it’s implementation can be found in the work of Ruf et al.^97^. The adopted spatial resolution was 7.5 $\mu$m in this experiment. Thus, the imaged region was approximately 14.5 mm wide and 11 mm high which allowed us to capture entire length of our sample columns.

Based on our setup, two different types of data acquisition protocols were realized during the EICP experiment (low-dosed) and the initial-/final step of the experiment (high-dosed) as shown in Table 1. In high-dosed data acquisition, the scanning data was acquired by collecting full-span projections (single projection per 0.25 degree of angle, 1440 projections in 360 degrees). In addition, this full-span data acquisition was performed five times while locating the detector at slightly different positions (shifting multiple pixels) at each batch of data acquisition. This was done to compensate for bad detectors pixels and to suppress inherent noise by averaging acquired projections at each acquisition angle (stitching cf.^97^). Note, that each batch of full-span acquisition took approximately 36 minutes thus, the total amount of the high-dosed scanning time including the image-improving stitching procedure took 3 hours (36 min $\times$ 5 $\approx$ 3 hours).

Given that the total amount of scanning time in our setup is mainly determined by the number of projections and the stitching procedure, we achieved a reduced data acquisition time by limiting them. Thus, in the low-dosed data acquisition, the stitching algorithm was not applied, despite aforementioned benefits, in order to reduce the total amount of scanning time. In addition, only 360 projections (single projection per each angle) were acquired which led us to significantly reduced scanning time (approximately 6 min per each batch of scanning). For both these acquisitions, we determined the shortest possible X-ray exposure time (500 [ms]) on our setup in order to further reduce the total scanning time.

**Table 1:** The detailed specifications of high- and low-dose scanning at each step.

	Projections	Exposure time [ms]	Stitching	Acquisition time	Experiment step
High-dose data	1440	500	Yes	3 h	Initial and final
Low-dose data	360	500	No	6 min	Intermediate

### Image post-processing

#### Reconstruction

The obtained projections with the aforementioned procedures in section $\mu$XRCT and Image acquisition were reconstructed with the commercial software Octopus Reconstruction^©^ (Version 8.9.4-64 bit) using the FBP method^64^. A simple beam-hardening correction and ring filtering method was applied which were supported by the software in order to cope with artefacts appearing in the scanned data after reconstruction. The original size of the reconstructed data, $1944 \times 1944 \times 1425$ voxels, was trimmed to the region of interest resulting in a final size of $1000 \times 1000 \times 1400$ voxels. Subsequently, the “imadjust” function in MatlabR2018a^©^^98^ was applied to enhance the contrast of the image for a better visibility of the features.

#### Image enhancement

**Figure 2:**
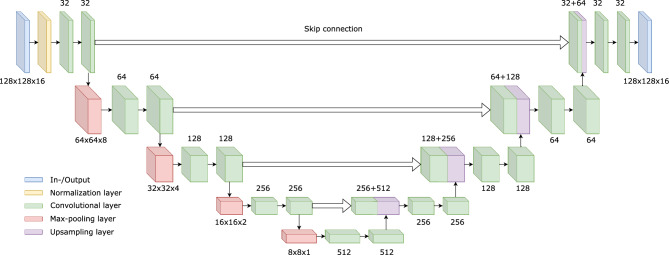
The architecture of the used 3D U-net. With the help of the convolutional, the max-pooling and upsampling layers, the model was designed to be optimized for the provided training data. The predicted output of the trained model is of identical size as the 3D images given as inputs (128 × 128 × 16).

In Fig. 2, the detailed architecture of our model is described. The standard structure of the 3D U-net model^99^ was adopted in order to enhance the low-dosed scanning data. The model is designed with sequential convolutional/max-pooling layers to down-sample features of the input. Later, the down-sampled information is up-sampled with corresponding convolutional/upsampling layers. The used size of the input is $128 \times 128 \times 16$. In our model, a batch normalization layer is used after the input in order to assist the generalization of our model during training as proposed by Zhou et al.^100^. Each resulting layer after the convolution and pooling operation and its corresponding up-sampled layer are concatenated by a skip connection. Each skip connection allows to use both the down- and up scaled features concurrently, which is beneficial to produce accurate predictions^101^.

Each convolutional layer is composed with a kernel size and activation function. The kernel size of the convolutional layer was chosen as $3 \times 3 \times 3$ with the “relu” activation function which leads its output range from 0 to $\infty$. The pool size of the pooling layers, i.e. max-pooling and up-sampling, was chosen as $2 \times 2 \times 2$ thus, the size of layers at each dimension is reduced by a half or increased by double compared to the previous layer after the operations. The size of the final output of the model is the same as the input size. The “sigmoid” activation function is applied at the final output layer which has its output range from 0 to 1.

The training data for our model was prepared artificially by selecting 360 raw projections (1 projection per 1 degree of rotational angle) from the complete 1440 projections (1 projection per 0.25 degree of rotational angle) and performing the reconstruction based on this subset with the FBP method. Note, that we adopted the raw projections before applying the stitching algorithm for the training data. These artificially created low-dose reconstructed images were later paired with the corresponding high-dose reconstructed images as a training set of our model. This combination of data was acquired at the final step, after the EICP process.

Further, only 30 slices of the total of 1400 slices of the reconstructed data of BGC1 were chosen to train the model. In order to avoid memory limitations during the training step, the chosen training data was cropped into small tiles with a size of $128 \times 128 \times 16$ voxels. In addition, each cropped tile contained overlapping regions to its neighbors (14 voxels at each side and 3 voxels at top/bottom) to cope with inaccuracies at the edges triggered by the convolutional operation^91^. In this way, the model was able to be trained to predict an enhanced reconstructed image from a noise affected low-dose reconstructed image.

During the training of our model, the Adam optimizer^102^ was adopted with the loss function “MSE” (Mean Squared Error). Details of the used training input parameters and specifications are displayed in Table 2. After the training, the cropped low-dose images with a size of $128 \times 128 \times 16$ were given as inputs to the trained model. The predicted outputs were later merged into the original images size ($1000 \times 1000 \times 1400$) after trimming out the overlapping regions. The model architecture and training was implemented with the help of the keras 2.3.1 library in python3.7. The implemented model was trained on a hardware consisting of Intel(R) Core(TM) i7-8750H CPU @ 2.2GHz, NVIDIA Quadro P1000 and 64 GB of RAM.

**Table 2:** Used training input parameters for adopted 3D U-net.

Variables	Parameters
Epochs	200
Used solver	Adam
Trainable parameters	2.3e + 7
Training time	5 h
Required memory	2.4 GB
Learning rate	8e − 5
Loss function	MSE

#### Segmentation

In order to evaluate the porosity of the sample and the precision of the prediction of the model, the segmentation process was performed allowing to differentiate between pore space and solid phase. This segmentation step was conducted using the “imquantize” function in MATLAB^©^^98^ which uses the intensity contrast within the image data. Since the method requires quantization levels for segmentation, these quantization levels were determined with the “multithresh” function which employs Otsu’s method^103^. Using the multi-level threshold approach was necessary to have a more accurate segmentation result compared to a single threshold approach, since the target image contained multiple features such as pore space, shrink tube, outer void and various solids (glass beads and calcium carbonate). Consequently, by using the function to differentiate the image in 4 classes (labelling the pixels 1–4 corresponding to their intensities) and collecting the pixels which were labelled bigger than 2, we were able to define the solid phase, e.g. the glass beads and the calcium carbonate as logical true and the rest, e.g. the pore space, shrink tube and the outer region, as logical false. Subsequently, the binarized images were trimmed as cylinders following the cross-sectional area of the scanned samples (masking). The radius of the mask was chosen as 340 pixels and centered to cover most of the region of interest. This is a simplification of the geometry, since the outer rim of the glass bead column is not perfectly cylindrical.

## Results

In the following, we present and discuss the results of the image enhancement using the U-net, including comparison to other image enhancement methods, the relation between pressure and porosity and the evaluation of the resulting pore-scale geometry.

### Result of image enhancement

**Figure 3:**
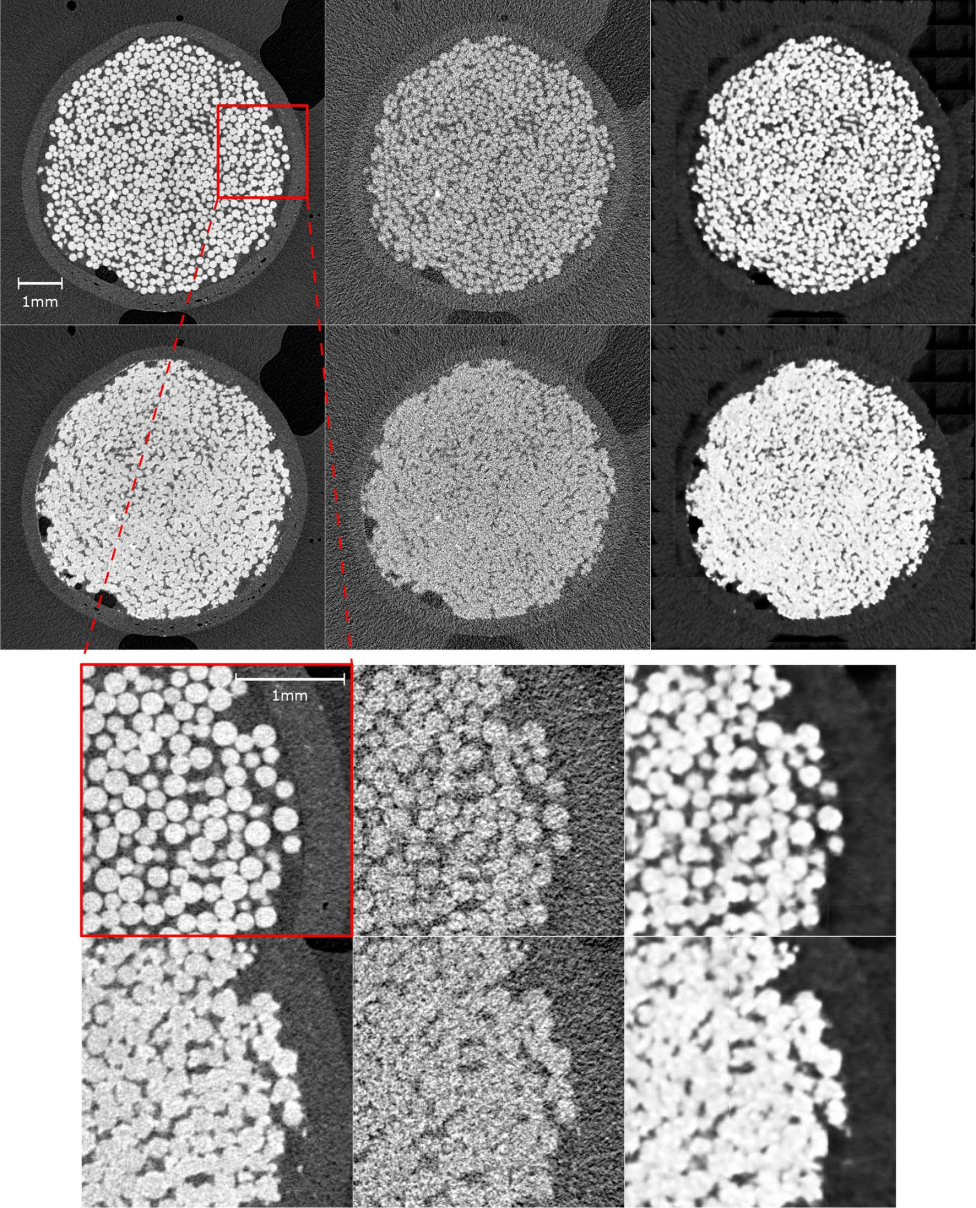
The 2D cross-sectional (x-y-plane, 662th slice of total of 1400 for BGC1) images of high-dose images (left), artificially created low-dose images (middle) and enhanced images by proposed workflow (right) are shown in corresponding locations at both the initial (top) and the final (bottom) stage. The region with red marker is magnified at the below accordingly.

**Figure 4:**
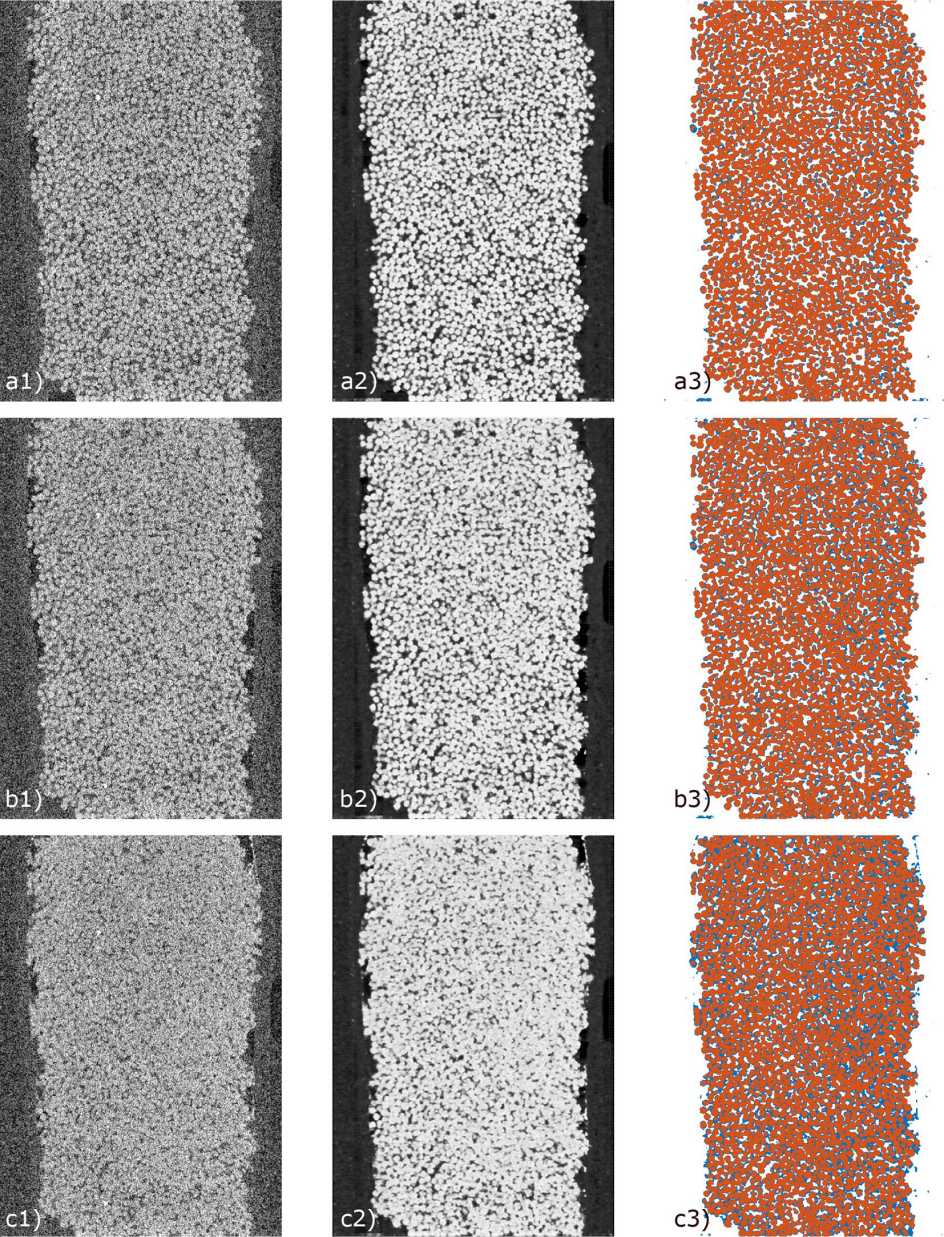
The 2D cross-sectional (x-z-plane, 500th slice of total of 1000 for BGC1) during EICP procedure. Representative images of the sample at different acquisition times ((**a**), (**b**) and (**c**) correspond to 1, 6 and 12 h). The images consist of low-dose images (**1**), corresponding predictions (**2**), and segmentations (**3**) (red: beads, blue: precipitation).

In Fig. 3, examples of the reconstructed data with high-/low-dose projections and their corresponding enhanced images obtained from our trained model are shown in order to demonstrate the effects of the enhancement. The shown images were not used in training. The images were chosen from the initial and the final time steps of the experiments, at which unprocessed images from high-dose scans are available for comparison. The low-dose images, which have noise and distorted feature structures due to the lack of information, were enhanced by applying our trained model. Specifically, the streaking artefacts and low signal-to-noise ratio in the low-dose images were improved significantly. In addition, the intensity contrast between solid parts and voids was increased which is beneficial to the subsequent image segmentation and further evaluations. We can observe a discontinuity of intensities at the edges and the top left part of the enhanced images, see Fig. 3, which are likely caused by the convolutional and pooling layers in our model. Potentially, this is due to the cropping of the images for training our model due to memory limitations, as described in section Image enhancement. A possible explanation is that the major portion of the used training data was taken from the inner part of the column without any part of the edge (e.g. the shrink tube or the epoxy mantle). Therefore, the discontinuity may be also triggered by a lack of training data. Nevertheless, the inner features where our main interest is on, were successfully enhanced with the provided training data set. Furthermore, detailed cross-sectional images can be found in Fig. 4. Our trained model can enhance and segment the poorly recognized features during EICP.

#### Validation of the image enhancement method

The predictive accuracy of our model was evaluated based on the segmentation of the model output (see section "Segmentation"). The reconstructed images of the high-dose (1440 projections, with stitching) and the low-dose projections (360 projections) were used in this evaluation. From the reconstructed images of the low-dose projections, our trained model produced the corresponding enhanced images. Subsequently, these enhanced images were segmented via following the workflow described in section "Segmentation". In order to minimize potential deviation due to the segmentation methods, the same segmentation workflow was followed for the enhanced low-dose and the high-dose reconstructed data. Eventually, the binarized images from the enhanced images and the high-dose reconstruction were compared with each other for the validation. The adopted accuracy estimation parameter was IOU (Intersection of Union) which is defined as:

4 $$ \begin{aligned} \text {IOU} = \frac{\text {Area of overlapping region (ground truth }  \& \text{ enhanced data )}}{\text {Area of Union (ground truth | enhanced data)}} \end{aligned}$$

where the ground truth is the segmentation results of the high-dose reconstruction and the enhanced data the output of the trained model with the low-dose images as inputs. Note that the full 3D binarized data (1000 $\times$ 1000 $\times 1400$ voxels) was used in this validation. The IOU, as described in Eq. (4), should give us “1”, if both images are identical or zero if there is no overlapping area. In Table 3, the resulting IOU’s of both experiments, BGC1 and BGC2, are greater than 0.89 for the final stage of our experiment, part of which was used for model training. For the initial stage of the experiments, the IOU are both greater than 0.82, even though none of the initial stage image data was used for training. (Readers interested in a detailed comparison of the pore size distribution histogram can refer to the Supplementary Information)

**Table 3:** IOU of predicted data at initial and final steps.

	BGC1	BGC2
Initial	0.8255	0.8265
Final	0.8947	0.8929

This accuracy gap between initial and final stage data could potentially be reduced by adding additionally data from the initial step to the training data set. However, in our study, we observed that the optimization process during the training of our model tended to diverge when training the model with both data from the initial and the final step, likely due to the increased complexity. Although the diverging tendency could be adjusted by reducing the learning rate, which is one of the hyperparameters responsible of updating the training model at each training step, this would cost increased training time due to reduced step size. Also, when using a tiny learning rate, the training process of the model often tends towards local minima or saddle point resulting in a non-generalized or under-trained model^104^. By taking this into account, the adopted model in our study was trained only with the data at the final stage after EICP process in order to prevent under-training and non-generalization. Also, it was important to apply a training data set which gives us stable training results, since the training data was cropped into small tiles in our study, which created a huge variety of training data, each tile of training data containing different information such as shape, contrast, and frequency of intensities, especially those tiles at the edges of the sample. Due to this variety of the training data, the optimization process of the model tended to either diverge or the training results to be too specific to a single tile of training data, when we applied the both data sets as a training data,

#### Comparison with different image enhancement methods

In the image enhancement process, we conducted a comparison using an additional ML-based model and an iterative reconstruction method to showcase the potential of the proposed method. Specifically, we chose the Generative Adversarial Network (GAN)^105^ and the Simultaneous Iterative Reconstruction Technique (SIRT)^106^ for this comparison. All of the methods employed in this comparison successfully enhanced the image quality. Among them, the U-net model, the main focus of this study, exhibited superior accuracy compared to others in terms of the IOU metric (SIRT: 0.8060, GAN: 0.8382, and U-net: 0.9058 in a representative volume of the acquired dataset after the final stage of the EICP experiment). Additionally, GAN demonstrated its capability to generate realistic images, while SIRT had the advantage of not requiring a pre-reconstructed image. In terms of memory and processing time, the ML-based models demanded fewer resources, with the U-net requiring less training time. For more details, interested readers can refer to the Supplementary Information.

### Relation between pressure and porosity

**Figure 5:**
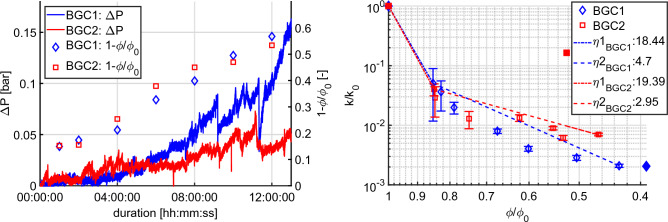
Left: Pressure difference ($\Delta {P}$) and $1 -\frac{\phi }{\phi _0}$ during continuous injection of both experiments, right: normalized permeability where *k* is permeability and $k_0$ is the permeability at the initial step. $\phi$ is porosity and $\phi _0$ is the initial porosity. The dashed lines indicate fitting curves corresponding to power-law in Eq. (2) with coefficients $\eta$. The permeability and porosity, measured before-/after the mineralization, are shown with the filled markers.

**Table 4:** Permeability of initial state ($k_0$) and after the precipitation ($k_{prec}$).

Experiment	$$\phi _0$$ [%]	$$\phi _f$$ [%]	$$k_0$$	$$k_{prec}$$ [−]	$$k_{prec} / k_0 $$ [−]	log10($$k_{prec} / k_0 $$) [−]
BGC1	29.50	11.56	2.55e − 11	5.26e − 14	0.002	− 2.68
BGC2	30.74	16.09	2.07e − 11	3.41e − 12	0.165	− 0.78

In Fig. 5, the monitored pressure differences between in-/outlet are displayed during the conducted experiments and the normalized porosities at each recorded time step are shown. The pressure difference ($\Delta {P}$) was measured during a constant flow rate of 1 $\mu {L}/s$. The porosity ($\phi$) was estimated from the binarized scanning data, following the procedure described in section "Segmentation". The inner part of the cylindrical mask, which was described in the section, was considered in this porosity estimation.

By taking into account that the initial porosity $\phi _0$ was from the high-dose scanning data in the shown figure, we observed a huge initial decrease in porosity within the first hour, especially when comparing to the other intervals. By considering the estimated accuracy of the model in Table 3, it is reasonable to speculate that the inaccuracy of the image enhancement caused this gap. A further hint towards this argument might be that the initial porosity reduction within the first hour is approximately 18 % of the initial porosity for both experiments. Additionally, this initial decrease in porosity might be triggered by the complex polymorphous nature of calcium carbonate precipitation. Often, metastable amorphous calcium carbonate (ACC) precipitates first, as a precurser of mineralization before transforming into crystalline CaCO$_3$^107,108^.

Due to its lower density compared to more stable and crystalline polymorphs vaterite or calcite, we believe the presence of ACC might influence scanning and its corresponding image enhancement results which requires further investigations^31^.

Based on the pressure measurement and porosity estimation, we also show the porosity and permeability relationship of the two experiments on the right side of Fig. 5. The permeability was computed by the procedure described in section "Experimental setup and procedure". The estimated permeability at the beginning of the injection has a relatively large error, defined by the standard deviation in this case. The reason for this is, that the pressure response of the sample at the early stage of the experiment contains rather low value-to-noise ratio and, therefore, the pressure response at the initial stage is not significant enough to be captured accurately. However, as mineralization proceeded during the experiment, the pressure drop increased and, thus, the reliability of the measured pressure data was also improved accordingly.

In order to enhance measurement reliability, as aforementioned in section "Experimental setup and procedure", the pressure drop of the sample before-/after the mineralization was measured for various flow rates. Consequently, based on the carefully measured initial permeability, the evolving intrinsic permeabilities observed during mineralization were normalized which showed degrading propensity as the porosity decreases.

The initial-/final porosity and permeability are shown in Table 4. Although both of the samples, BGC1 and BGC2, have comparable initial porosity and permeability, the final reduction of permeability was not identical. In the approximated power-law relation (Eq. (2)) fitted to the measured porosities and permeabilities, the exponents $\eta$ after 1 hour were estimated as $\eta {2}_{BGC1} = 4.7$ and $\eta {2}_{BGC2} = 2.95$, which means that they are in the range of reported values ($\eta \approx {}$2 to 10) in literature^30^. The exponents before 1 hour, however, were relatively large ($\eta {1}_{BGC1} = 18.44$ and $\eta {1}_{BGC2} = 19.39$). A significant drop of permeability at the beginning phase of an ICP application was also observed in previous experimental studies^31,109,110^. Based on the previous investigations, we believe that the measurement with the estimated porosity from the enhanced scan data showed a good agreement with previous studies. The final porosity and permeability of BGC2 show an irregular tendency in Fig. 5. Based on a careful interpretation of the scan data, we believe the unexpected behavior was caused by the flushing procedure, conducted to prevent further mineralization before the final scanning (see section "Experimental setup and procedure").

## Conclusion

With our proposed workflow, we were able to observe porosity changes in 4D caused by the evolution of mineralization with EICP. Low-dosed projections, by taking 1 projection per an angle and reduced total exposure time (no-stitching), were collected during the EICP process in order to reduce data acquisition time (from 3 hours down to 6 min) to a time period short enough to neglect changes triggered by calcium carbonate precipitation within the imaging time. Subsequently, the low-dose data was reconstructed with the FBP method. The resulting quality degradation due to the low-dose strategy, was enhanced with an adopted machine learning model (3D U-net) for further evaluation.

In order to deal with the large amount of data in an efficient way (7 low-dosed datasets, each consisting of $1000 \times 1000 \times 1400$ voxels), we adopted the 3D U-net model and FBP reconstruction method instead of more complex machine learning models that require high computational costs and innate memory. The model was trained to map a feature from a low-dose to a high-dose reconstruction where the high-dose data were acquired after the EICP process. The model which was trained with only a few subsets of the data (30 slices), was able to effectively reduce both noise and artefacts while conventional filtering approaches are considered to have difficulties with these noises and artifacts within a reconstructed image^111^. The accuracy of enhanced images by our trained model was evaluated by comparing them to high-dose data with high fidelity. With the help of binarization, we showed that the estimated porosities from the enhanced images were in the range of the high-fidelity data which were acquired before-/after mineralization by EICP. From the increasing measured pressure differences between in-/outlet of each sample during the EICP process, we infer a decrease in permeability caused by mineralization.

Based on the presented results, we expect that the proposed workflow can be used for further investigation of mineral precipitation in porous media and, especially, the detailed investigation of realistic porosity-permeability relations for various relevant porous media and mineral precipitation conditions. Using 3D samples instead of 2D samples^21,31–34^, the determined porosity-permeability relations can be expected to be more realistic than those obtained from the 1D or 2D setups in the mentioned references, as 3D systems allow for pore connections in all three dimensions and, at least for our investigation, permeability decrease seemed to a large part to be controlled by the connectivity or rather disconnection of pores. However, we also want to stress that to determine realistic porosity-permeability relations, great care has to be taken for the sample to be representative for the porous medium investigated. For example, our sample BGC2 had a clear preferential flow path at the edge of the sample, increasing the apparent permeability of BCG2 thereby reducing the information on the effects of porous-medium mineralization that can be gained. Additionally, evaluating the changes of individual pore bodies over time, we demonstrated that while the total number of pore bodies does not change much during mineralization in our glass-bead columns, more and more pore bodies got disconnected as mineralization progressed. Further, we noticed in this evaluation that sample BGC1 had more homogeneously distributed pore bodies while BGC2 had a clear preferential flow path. Those features were preserved during mineralization and seem to be important for the degree of permeability reduction due to EICP (interested reader refers to Supplementary Information). In future, the accuracy comparison with data obtained at a synchrotron light source could be used to investigate the suspected effect of ACC on the accuracy of our workflow and especially the early-stage of the porosity estimation.

## Supplementary Information

Supplementary Information.

## Data Availability

The measured pressure and acquired tomograhpic data during conducted experiment are available at DARUS, *Time resolved micro-XRCT dataset of Enzymatically Induced Calcite Precipitation (EICP) in sintered glass bead columns* via doi:10.18419/darus-2227 under no registration and create commons attribution conditions.

## Code availability

The first version of *Image enhancement code: time-resolved tomograms of EICP application using 3D U-net* used for enhancing the low-dose images is preserved at doi:10.18419/darus-2991 under no registration and create commons attribution conditions. The code is developed in Python (https://www.python.org/).
