# Supplementary material for: Machine learning assists in increasing the time resolution of X-ray computed tomography applied to mineral precipitation in porous media
Source: Sci Rep. 2023 Jun 29;13:10529. doi: 10.1038/s41598-023-37523-0 (PMC10310824; doi:10.1038/s41598-023-37523-0)
Supplement: Supplementary file 1 — Supplementary Information. [file 41598_2023_37523_MOESM1_ESM.pdf]

Supplementary Information: Machine Learning assists in increasing the time resolution of X-Ray Computed Tomography applied to mineral precipitation in porous media

Dongwon Lee[1,*], Felix Weinhardt[2], Johannes Hommel[2], Joseph Piotrowski[3], Holger Class[2], and Holger Steeb[1,4]

[1]Institute of Applied Mechanics (CE), Pfaffenwaldring 7, University of Stuttgart, 70569, Germany
[2]Institute for Modelling Hydraulic and Environmental Systems, Pfaffenwaldring 61, University of Stuttgart, 70569 Stuttgart, Germany
[3]Agrosphere (IBG-3), Institute of Bio- and Geosciences, Forschungszentrum Jülich, 52425 Jülich, Germany
[4]SC SimTech, University of Stuttgart, Pfaffenwaldring 5, Stuttgart, 70569, Germany
[*]dongwon.lee@mechbau.uni-stuttgart.de

Damköhler number and Péclet number

The Damköhler number[1] indicates in our study that reaction is much slower than transport, and it reduces from initially 0.014 to 0.002. Péclet numbers[2] similarly decrease over the course of the experiment from 96 to 38.

Characteristic reaction time

The characteristic time scale of induced carbonate precipitation (ICP) at the conditions in our study is approximately 5000 s, as estimated using the kinetic rate equations from[3]. Thus, a 6 min XRCT scanning time for the low-dose scans as well as a sampling interval of 1 h is fast compared to the expected change in the volume of precipitates. However, the high-dose XRCT scans pre- and post-mineralization took approximately 3 h, which would likely not sufficiently resolve the ICP process in our samples.

Kozeny-Carman relation versus power-law relations

Kozeny-Carman type porosity-permeability relations are commonly used by many authors for describing the changes in permeability due to ICP[4,5]. They, and other sophisticated porosity-permeability models, can be superior to simple power laws if information about the specific geometry change in the pore space is available and understood[6]. Power laws are simple fitting approaches which are easy to intercompare with results of others. We decided to use the power-law relation to be able to compare the impact of ICP on the porosity-permeability relation of the column samples to other studies, such as Weinhardt et al.[7], who published power-law fittings due to ICP for 1D and 2D microfluidic systems. Many Kozeny-Carman type relations used for ICP scenarios use simplifying assumption, e.g. assuming negligible changes in particle shape or pore geometry[6]. Thus, in order to apply a Kozeny-Carman type relation properly and with justification of the more sophisticated approach, it requires to rather define the change of parameters such as particle diameter and shape or porous-medium tortuosity due to ICP, which are (i) not commonly available in REV-scale models and (ii) would introduce further intermediate parameters. While the tortuosity or particle diameters and shapes could be easily determined in an experimental study using imaging such as ours, for modeling the ICP process in porous media, this would result in splitting the porosity-permeability relation into e.g. a porosity-tortuosity and a tortuosity-permeability relation, thereby artificially increasing model complexity.

Pore body evaluation

In this section, the image processing procedure in order to extract pore sizes is described. The pore bodies of the used sample during the experiments were evaluated at each time step. The low-dose reconstruction images acquired at each time step were enhanced with the trained model and segmented via the aforementioned workflow. Based on these enhanced and binarized images, the watershed method[8] was applied in order to define each individual pore body. The method identifies connected volume as “basins” (pore bodies) using a distance map or intensity gradients. This procedure was performed in MATLAB R2018a[9]. The detailed workflow is as follows:

(1st) The binarized images were trimmed as cylinders following the cross-sectional area of the scanned samples (masking). The radius of the mask was chosen as 340 pixels and centered to cover most of the region of interest. This is a simplification of the geometry, since the outer rim of the glass bead column is not perfectly cylindrical. (2nd) Isolated voxels, which may cause inconsistencies within the final output, were removed with the help of the “bwmorph” function with the “majority” option. (3rd) In order to separate the pores, we computed a distance map which provides a higher value for larger space and lower value for smaller space.

Therefore, voxels in the pore bodies were assigned higher values while the narrow adjacent pore throats were assigned lower values. The distance map was computed with the “bwdist” function and the “city-block” option. (4th) In order to prevent the well-known over-segmentation issue with the watershed method[10], the 3D median filter[11] was applied to the computed distance map which helped to reduce the segmentation sensitivity. (5th) The inverse operation was conducted to the treated distance map. Thus, the output of this operation could be used as a basin. (6th) Finally, the “watershed” function was applied so that the individual basins were differently labelled. Subsequently, the rest, which are “non-pores”, including solid parts and exteriors of masked area, were defined as zero. Therefore, only the interior “pores” were considered in further volume estimation.

Comparison of pore sizes between high-dose images and model output

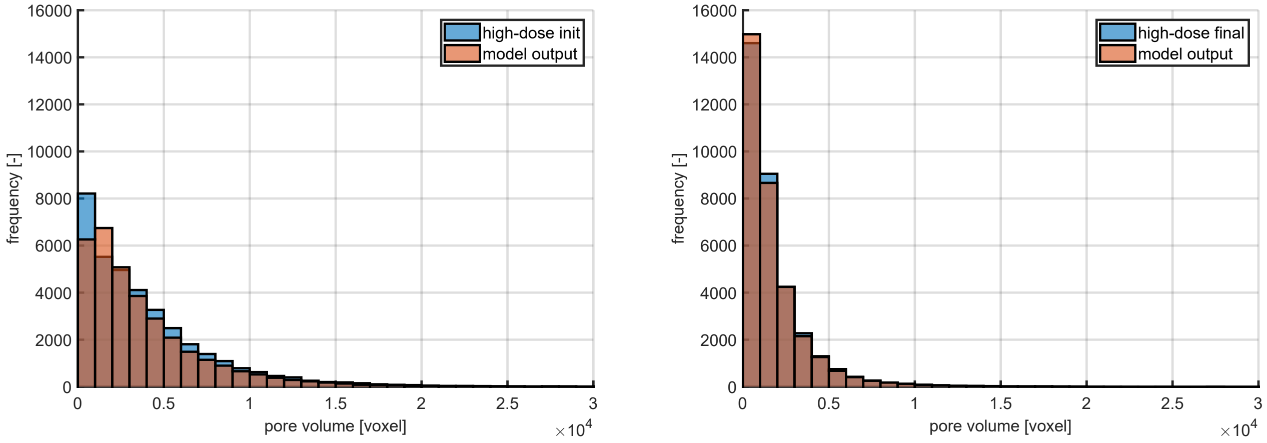

Figure 1. The comparison of pore sizes was conducted between high-dose images and improved images, and the pore sizes were extracted at both the initial (left) and final (right) stages of the experiment.

To provide a more detailed explanation of model validation, we performed further comparison of pore sizes. As quantified by the Intersection over Union (IOU) metric in Validation of the image enhancement method, the histogram showed a good match in the final state but a poor match in the initial state of the experiment. In particular, at the initial state of the experiment, the smallest pore sizes were not well recognized, and their sizes were slightly overestimated due to inaccuracies in the model. However, pore sizes larger than 2000 voxels (where the pore radius is estimated to be larger than 59 μm) exhibited a relatively small deviation.

Geometry alterations on the pore scale

In Figure 2, histograms of the evaluated radii of the volume of pore bodies are shown. The pore bodies were identified following the procedure described in section Pore body evaluation. Subsequently, the separated pore bodies were approximated as spheres with corresponding radii. We defined the connected pore bodies by excluding pore bodies which are not connected to the inlet and outlet and therefore do not contribute to flow.

Note that the total number of pore bodies was comparable at each time step after following the procedure described in section Pore body evaluation. Thus, the results of the segmentation were consistent (the total number of all pore bodies in BGC1: 32582, 32625, 32263 and in BGC2: 30848, 30895, 29224 at each selected time step 1,6,12 hours). This indicates that we did not have a significant over-segmentation problem which may be issued by using the adopted watershed segmentation method. However, comparing the connected pores for each time step, the total numbers decreased as disconnection occurred (the total number of connected pore bodies in BGC1: 32475, 31839, 21442 and in BGC2: 30578, 29379, 21455 at each selected time step 1, 6, 12 hours).

In general, both histograms, the one of all pore body sizes and the one of connected pore body sizes, show that the size of the pore radii decreases during mineralization. Especially, the larger pore bodies, whose initial radius exceeded 20 pixels,

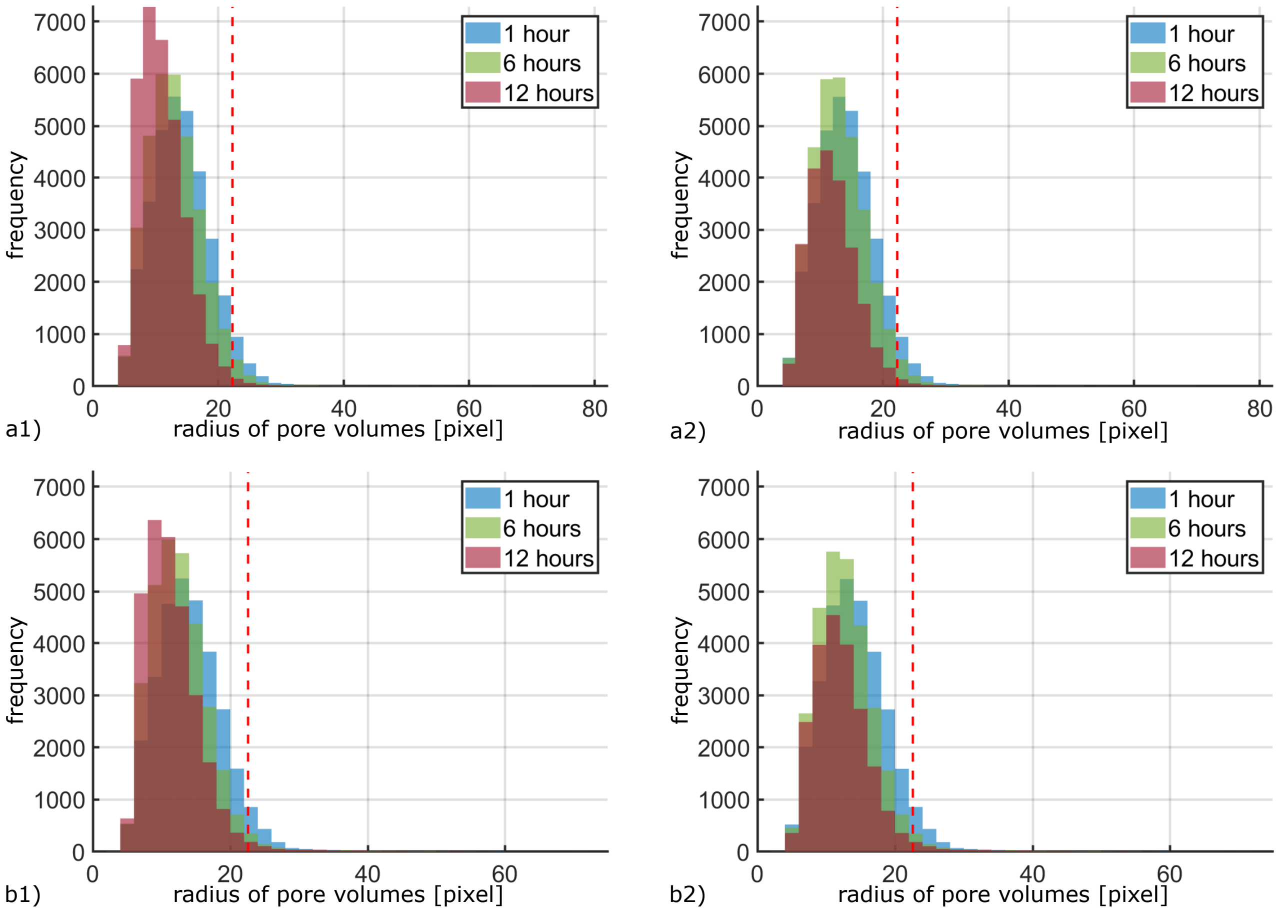

Figure 2. The pore radii of BGC1 a) and BGC2 b). The pore radii of all pore bodies a1,b1). The pore radii of connected pore bodies a2,b2). The dashed red line indicates 95 % quantile of the 1 hour histogram

decreased in size during the experiment. The histograms of the connected pore bodies (a2 and b2) show that the numbers of connected pore bodies were reduced over time. The difference at the later stage, between 6 and 12 hours, was significant. This seems to be caused by two possible scenarios: (1st) The reduction of pore body sizes became significant after a certain amount of mineralization causing some pore throats to clog, disconnecting pore bodies. 2nd The pore body sizes became too small to be resolved with the chosen spatial resolution of XRCT (7.5 μm/voxel) so that those were treated as disconnections.

Given that there was no significant change in the median radii of the connected pore bodies during the experiment, we can infer that the disconnection of pore bodies likely had a critical impact on the observed reduction in permeability of the samples. In addition, by the comparison between the histograms of all and connected pore bodies only, we observed that the smaller pore bodies tend to disconnect more often.

Figure 3 shows the connected pore bodies larger than the 0.95 quantile of all pores after 1h (indicated with red dashed line in Figure 2) at time steps of 1, 6, and 12 hours in 3D. The many small pore bodies are omitted for the sake of a better visualization of the large pore bodies likely dominating the measured permeability of the samples. The color scheme was applied in order to emphasize the pore-body sizes. The larger pore bodies are colored in orange and the smaller ones in blue. Both samples show a reduction of the large pore-body sizes. Especially, pore bodies with pore radii of approximately 20 to 65 were remarkably reduced in number, while the number of the largest pore bodies did not change significantly.

Despite of the comparable initial porosity of both sample BGC1 and BGC2, the pore morphology of the samples were different (see Figure 3, BGC2 had an initial preferential flow path). In the sample BGC1, the mineralization was homogeneous, while the mineralization of the sample BGC2 did not clog the initial preferential flow path, as the group of large-sized pores at its side remained until the end of the mineralization. This could explain the variation in permeability reduction between the two samples. The permeability reduction of BGC1 was more significant and stable during the experiment due to the homogeneous decrease of the number of larger pores. In contrast, for experiment BGC2, the preferential flow path still remained open during

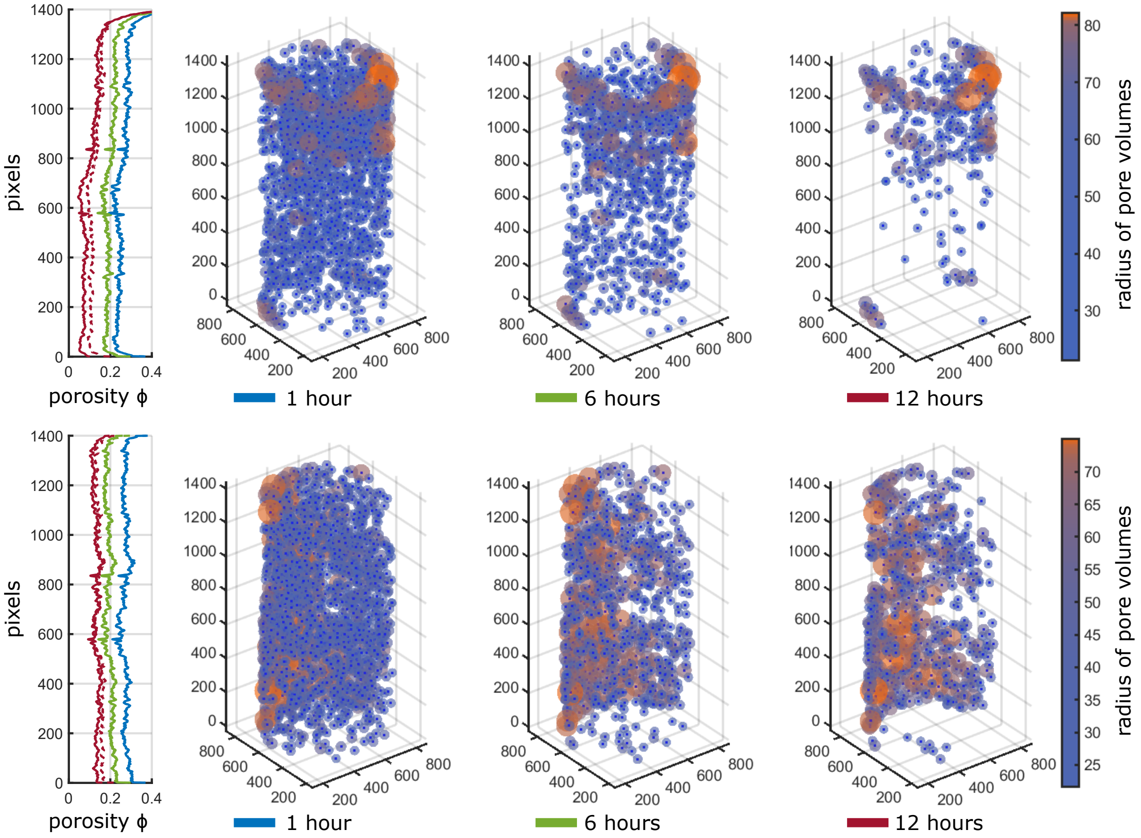

Figure 3. The 3D visualization of connected pore bodies during the experiment (top: BGC1, bottom: BGC2). The general porosity is marked with dashed line and the porosity of connected pores only is marked with filled line. The injection of solutions was conducted from the top part (1400 pixel).

the mineralization. This is in agreement with observations described in the work of Weinhardt et al.[7]. In addition, it is possible that the remaining, only partly clogged preferential flow path caused the increased final permeability of BGC2 after flushing. Since there was a preferential flow path at the side, the mineralization at the rest of domain would not contribute much to permeability reduction since the remaining preferential path would still dominate the sample's overall resistance to fluid flow.

Generative Adversarial Network (GAN)

In our comparison, a simple structure of GAN[12] model was adopted. For the generator of the GAN, we adopted the same structure as the U-net model described in our main manuscript. As for the discriminator, we utilized a sequential architecture with convolutional layers and a "relu" activation function. The activation function at the output layer of the discriminator was set to "sigmoid," which was the same as the activation function used in the used U-net model. The schematic of the discriminator can be found in Figure 4. The discriminator was trained in two scenarios: one with a pair of low-dose and high-dose data along with real labels, and the other with a pair of low-dose and generated data along with fake labels. This training approach enabled the model to learn to distinguish between high-dose images and the 'real-like' generated images. The same training data that was used to train our proposed U-net model was also utilized to train both the generator and discriminator of the GAN. The specific parameters employed in the training of the GAN can be found in Table 1.

Simultaneous Iterative Reconstruction Technique (SIRT)

The SIRT[13] used in this study is an iterative reconstruction method. It begins with an initial guess of the reconstructed volume. The algorithm then computes forward projections based on the current estimated reconstruction volume. These forward projections are compared with the acquired projections to evaluate the difference. The algorithm backprojects these differences into the reconstructed volume and updates the current estimate by adding the backprojected differences. This process is repeated until convergence is achieved, refining the estimated shape of the scanned features. As the number of iterations increases, the algorithm progressively improves the accuracy of the estimated shape of the scanned data. Despite the great potential of the

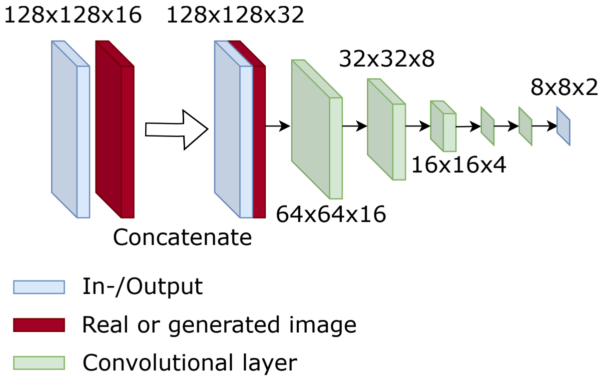

Figure 4. The architecture of the discriminator during training of the used GAN.

Variables	Parameters	
	Generator	Discriminator
Epochs	200	
Used solver	Adam	
Trainable parameters	2.3e+7	2.9e+6
Training time	20 hours	
Required memory	3.1 GB	
Learning rate	8e-5	
Loss function	MSE	Binary Crossentropy

Table 1. Used training input parameters for adopted 3D GAN

method to enhance under-sampled data, it is well-known for its high computational demands[14]. Due to the computational demands of the method, a subvolume size of $1000 \times 1000 \times 300$ voxels was selected for this study. Specifically, the middle part of the whole stack was chosen and extracted to reduce the computational burden.

As depicted in Figure 5, there is an improvement in the visualization quality as the number of iterations increases. Specifically, previously blurry features that made it difficult to discern the boundaries between the solid and void phases have become more recognizable.

To address the remaining ring artifacts, a 2-D order statistic filter[15] was utilized. This filter replaces the intensities within the ring-shaped region (or a customized domain shape) with the median intensity of its neighboring pixels. By applying this filter, the ring artifacts are effectively mitigated, resulting in improved image quality.

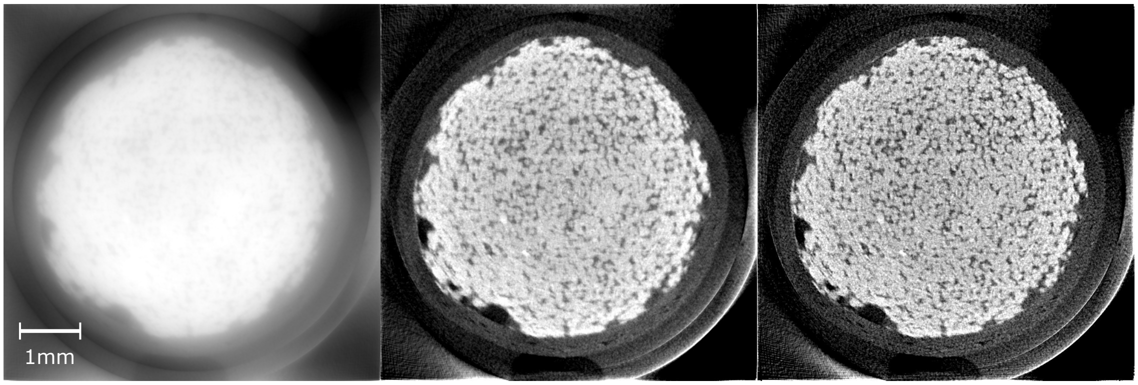

Figure 5. The example 2D cross-sectional images were generated by applying the SIRT algorithm with different iterations. The images from left to right correspond to iterations of 5, 150, and 500, respectively.

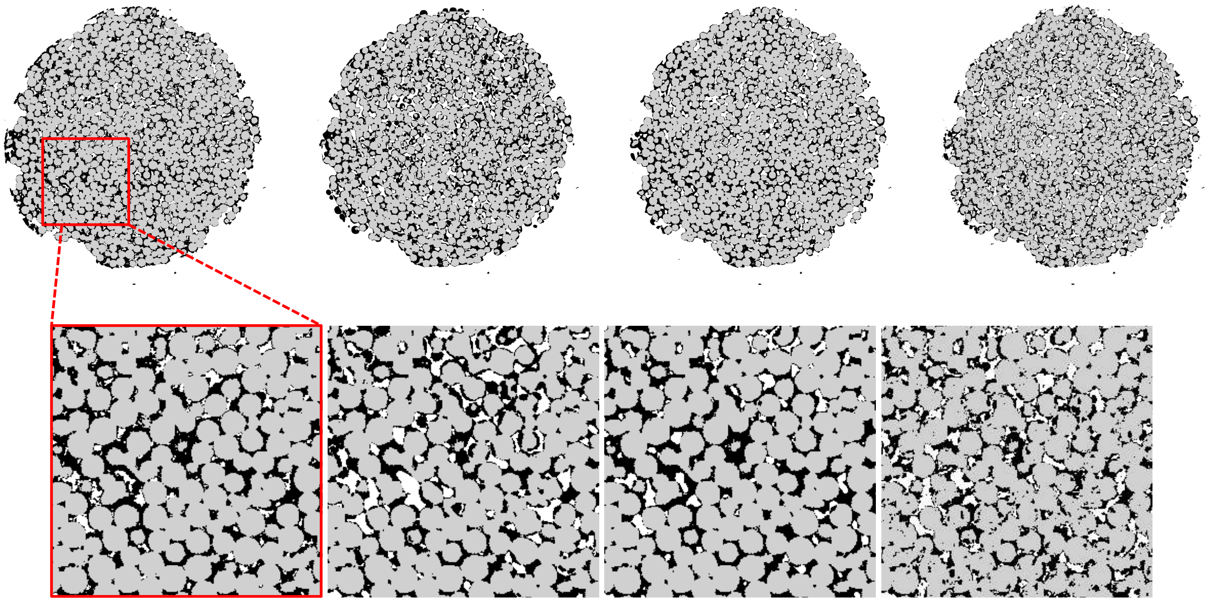

Figure 6. The segmentation example of 2D cross-sectional images (x-y plane, 622th slice out of a total of 1400 for BGC1) at the final stage of the EICP experiment is shown. In the images from left to right: high-dose (GT), SIRT, U-net and GAN images. The black marked area represents precipitation, while the gray marked area represents glass beads. The region marked in red is magnified accordingly for better visibility.

Segmentation results with Intersection of Union (IOU)

In the comparison of the improved images obtained through the adopted image enhancement methods, the images were segmented into three phases: glass beads, precipitation, and void. For the segmentation, the image was classified into two phases: solids and voids. Subsequently, the segmented glass beads obtained from the high-dose image in the initial stage before the EICP experiment were used as a mask to discriminate between precipitation and glass beads. The segmentation quality was evaluated and compared among the methods. For this comparison, the final dataset of BGC1 was used as a subvolume comprising slices 569th to 856th, totaling 288 slices out of the total of 1400 slices. This subvolume was selected due to the memory constraints associated with the SIRT method. Note that 22 slices out of the initially chosen 300 slices were not evaluated in this segmentation study, as SIRT failed to resolve those slices located close to the upper and lower boundaries of the subvolume.

Since the segmentation method can also affect the comparison results, we employed a simple segmentation method to ensure a fair comparison. The multithreshold method (Otsu) was applied to the enhanced images obtained from the adopted image enhancement methods. The segmentation results are presented in Figure 6. Furthermore, the IOU (Intersection over Union) metrics were calculated for the segmented images using the same subvolume of 288 slices (from 569th to 856th). The IOU values obtained for each method are as follows: SIRT: 0.8060, U-net: 0.9058, and GAN: 0.8382. Based on these results, it is evident that the Unet model outperformed the other methods in terms of segmentation accuracy, as it achieved the highest IOU value for the subvolume.

Comparative analysis of image enhancement methods

To aid in comprehending the performance and potential of the proposed method, we employed additional image enhancement techniques and conducted a comparison. For this comparison, we utilized two ML-based models: the U-net, which is our proposed model in this study, and the GAN (Generative Adversarial Network)[12]. Furthermore, we adopted the SIRT (Simultaneous Iterative Reconstruction Technique)[13], an iterative reconstruction algorithm supported by ASTRA toolbox[16]. The SIRT result in this study was obtained after 500 iterations. The detailed descriptions and explanations of the adopted methods can be found in the Supplementary Information.

As shown in Figure 7, all of the adopted methods successfully enhanced the visualization quality, aiding in the differentiation between the pores and solids. Among the enhanced outputs, the U-net yielded a distinct boundary between the different phases. The GAN model generated an image that appeared more realistic compared to the U-net for the given low-dose image. This observation align with the inherent nature of our used GAN model, which focuses on generating realistic images. Unlike to the

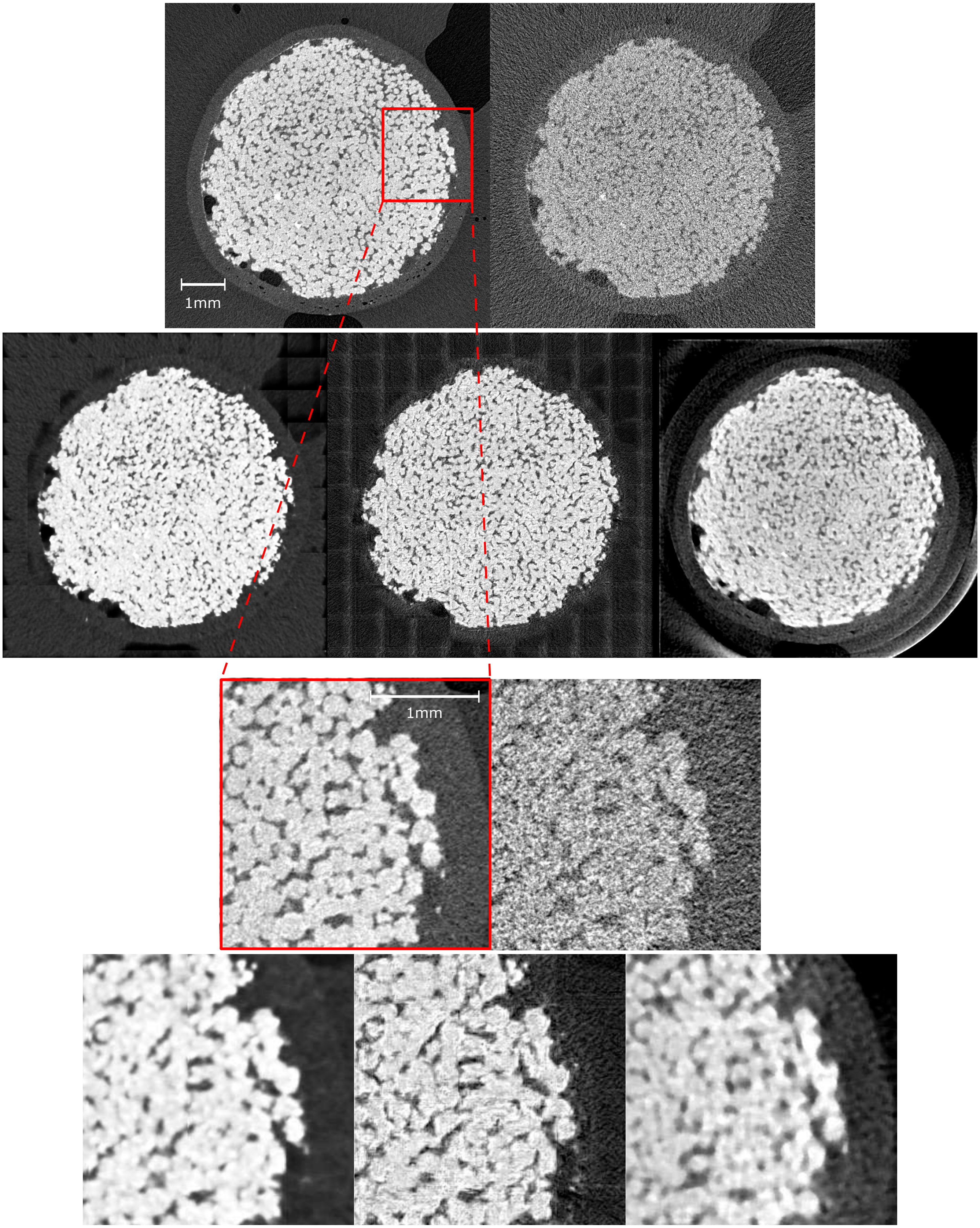

Figure 7. The 2D cross-sectional (x-y-plane, 662th slice of total of 1400 for BGC1) images were produced using different means. On the top, the high-dose (left) and low-dosed (right) images are demonstrated. Below, the enhanced images generated by the U-net, GAN and SIRT algorithm are displayed from left to right. The region marked in red is magnified correspondingly in each image.

outcomes of the both ML-based models, the image enhanced by the SIRT exhibited blurry phase boundaries and noise-related issues, despite the use of relatively large number of iterations.

The result of GAN, which contains realistic noises, can be attributed to the characteristic of GAN. The GAN model contains two types of models: the generator and the discriminator. The generator is trained to produce an enhanced image based on a given low-dose image, while the discriminator is trained to distinguish between "real" and "fake" images. During GAN training, the generator produces an enhanced image that aims to resemble a "real-like" image while the discriminator evaluates the generated image and determines whether it is "fake" or "real". The decision of the discriminator serves as feedback to guide the models in reducing their losses. Consequently, the generator is trained to create a more "real-like" image that can deceive the discriminator. Due to this characteristic, the enhanced image produced by the GAN model may contain inherent noise that was present in the high-dose image.

In the SIRT result, the visualization quality is significantly improved compared to the low-dose image. However, it is observed that the reconstructed features near the outer region suffer from blurriness and distortion. This is primarily due to the limited information available in the acquired projections, especially at the edges. These defects can be mitigated by providing a more accurate initial guess or increasing the number of iterations, as the method aims to enhance its image quality by reducing the disparity between the acquired projection and the estimated projection based on the assumed reconstruction volume

The computation time of each adopted method was also evaluated. For this comparison, a subvolume of $1000 \times 1000 \times 300$ voxels was considered for simplicity. The subvolume was extracted from the middle part of BGC1 in the final stage after the EICP experiment. There was no significant difference in the processing time between the ML-based models. Both models took approximately 8 minutes. However, the training time of the GAN model was much longer compared to the U-net. The GAN training took approximately 20 hours, while the U-net training was completed in around 5 hours. This is because the GAN model requires optimizing the loss for both the generator and discriminator. Additionally, the GAN model necessitated additional memory for the discriminator model. In the case of SIRT, the algorithm required 6 GB of GPU memory for the given subvolume. As a result, the investigation had to be performed on a different device with a more powerful GPU, specifically a CPU Intel(R) Core(TM) i7-8750H CPU @2.2GHz and a GPU NVIDIA GeForce GTX 1080 Ti. On this machine, the SIRT algorithm took approximately 20 minutes for 500 iterations.

In this comparison, the ML-based models showcased superior performance in terms of output quality and computation time compared to the adopted iterative reconstruction method. Moreover, when considering accuracy as measured by the IOU metric, the U-net model outperformed the other methods (SIRT: 0.8060, GAN 0.8382 and U-net: 0.9058). The GAN model was capable of generating realistic data, but the embedded noise in its results posed challenges for subsequent segmentation steps. However, by modifying the training data, such as pairing low-dose and high-dose filtered images, the output quality for further segmentation could potentially be improved. Additionally, the GAN model had longer training times and required larger memory resources. We expect that these trends would likely hold for more sophisticated ML models. Despite these issues, we have shown that our approach is applicable to different types of ML models. There is still room for further research to determine which types of models would perform best in this approach, especially when dealing with more complex data. Moreover, it is important to note that the SIRT algorithm, which does not require training or preconditions for reconstruction, also holds great potential for further investigations.

References

1. Fogler, H. S. *Elements of chemical reaction engineering (5th)* (Prentice Hall, 2021).
2. Rapp, B. E. Chapter 9 - fluids. In Rapp, B. E. (ed.) *Microfluidics: Modelling, Mechanics and Mathematics*, Micro and Nano Technologies, 243–263, DOI: https://doi.org/10.1016/B978-1-4557-3141-1.50009-5 (Elsevier, Oxford, 2017).
3. Hommel, J. *et al.* A numerical model for enzymatically induced calcium carbonate precipitation. *Appl. Sci. (Switzerland)* **10**, 1–26, DOI: 10.3390/app10134538 (2020).
4. van Wijngaarden, W. K., Vermolen, F. J., Meurs, G. A. M. & Vuik, C. A mathematical model for Biogrout. *Comput. Geosci.* **17**, 463–478, DOI: 10.1007/s10596-012-9316-0 (2013).
5. Yasuhara, H., Neupane, D., Hayashi, K. & Okamura, M. Experiments and predictions of physical properties of sand cemented by enzymatically-induced carbonate precipitation. *Soils Foundations* **52**, 539–549, DOI: 10.1016/j.sandf.2012.05.011 (2012).
6. Hommel, J., Coltman, E. & Class, H. Porosity-permeability relations for evolving pore space: A review with a focus on (bio-)geochemically altered porous media. *Transp. Porous Media* **124**, 589–629, DOI: 10.1007/s11242-018-1086-2 (2018).
7. Weinhardt, F. *et al.* Spatio-temporal distribution of precipitates and mineral phase transition during biomineralization affect porosity-permeability relationships - Microfluidic investigations. *Transp. Porous Media* **143**, 527–549, DOI: 10.1007/s11242-022-01782-8 (2022).

8. Meyer, F. Topographic distance and watershed lines. *Signal Process.* **38**, 113–125, DOI: 10.1016/0165-1684(94)90060-4 (1994).

9. MATLAB. *(R2018a)* (The MathWorks Inc., Natick, Massachusetts, 2018).

10. Thomson, P.-R., Aituar-Zhakupova, A. & Hier-Majumder, S. Image segmentation and analysis of pore network geometry in two natural sandstones. *Front. Earth Sci.* **6**, DOI: 10.3389/feart.2018.00058 (2018).

11. Huang, T., Yang, G. & Tang, G. A fast two-dimensional median filtering algorithm. *IEEE Transactions on Acoust. Speech, Signal Process.* **27**, 13–18, DOI: 10.1109/TASSP.1979.1163188 (1979).

12. Goodfellow, I. *et al.* Generative adversarial nets. In *Advances in neural information processing systems*, 2672–2680 (2014).

13. Gilbert, P. Iterative methods for the three-dimensional reconstruction of an object from projections. *J Theor Biol* **36**, 105–117 (1972).

14. Beister, M., Kolditz, D. & Kalender, W. A. Iterative reconstruction methods in x-ray ct. *Phys. Medica* **28**, 94–108, DOI: https://doi.org/10.1016/j.ejmp.2012.01.003 (2012).

15. Huang, T., Yang, G. & Tang, G. A fast two-dimensional median filtering algorithm. *IEEE Transactions on Acoust. Speech, Signal Process.* **27**, 13–18, DOI: 10.1109/TASSP.1979.1163188 (1979).

16. van Aarle, W. *et al.* Fast and flexible x-ray tomography using the astra toolbox. *Opt. Express* **24**, 25129–25147, DOI: 10.1364/OE.24.025129 (2016).
